# Female Athlete Triad Awareness Among Multispecialty Physicians

**DOI:** 10.1186/s40798-015-0037-5

**Published:** 2015-11-12

**Authors:** Emily J. Curry, Catherine Logan, Kathryn Ackerman, Kelly C. McInnis, Elizabeth G. Matzkin

**Affiliations:** 1 Women’s Sports Medicine, Department of Orthopaedic Surgery, Brigham and Women’s Hospital, Boston, MA USA; 2 Division of Sports Medicine, Boston Children’s Hospital, Boston, MA USA; 3 Physical Medicine and Rehabilitation, Massachusetts General Hospital, Boston, MA USA; 4 Harvard Medical School, Boston, MA 02115 USA

## Abstract

**Background:**

The female athlete triad (Triad) is a serious condition with lifelong consequences seen in physically active females. Prior studies assessing Triad knowledge among coaches/athletic trainers reported surprisingly low awareness results. Our aims were to (1) determine the percentage of physicians across multiple specialties who had heard of the phrase “female athlete triad” and (2) determine the percentage who can properly diagnose or have a high comfort level appropriately referring these patients.

**Methods:**

Via electronic survey, we recruited medical staff, residents, and fellows at three large academic institutions across specialties to answer an eight-item test on Triad awareness and knowledge.

**Results:**

A total of 931 physician participants were recorded. Of the total responders (40 % male and 60 % female), 23 % were residents, 12 % were fellows, and 65 % were attending physicians. Overall, 37 % had heard of the Triad. Of these respondents, an average of 2.1 ± 1.1 of the three components were properly identified with an overall average score on the Triad awareness test of 71 ± 18 % out of a possible 100 %. Fifty-one percent reported feeling either comfortable treating or referring a patient with the Triad.

When assessing awareness among specialties, the awareness rates were highest among orthopedic surgery (80 %), followed by obstetrics and gynecology (55 %) and physical medicine and rehabilitation/rheumatology (52 %). The three with the lowest awareness were anesthesia (9 %), radiology (10 %), and psychiatry (11 %).

**Conclusions:**

Our findings suggest that approximately one third of the physicians surveyed have heard of the Triad. Approximately one half of physicians were comfortable treating or referring a patient with the Triad. Increased awareness through education to properly identify and manage the Triad is essential for all physicians.

## Key Points

- There is low awareness of the female athlete triad among physicians across specialties.
- It is likely that many females are not diagnosed and subsequently treated for the Triad given the high number of physician responders who were unable to properly identify the Triad in a clinical setting.
- Our results suggest that major effort is needed to increase awareness of the Triad across medical and surgical subspecialties.

## Background

After Title IX of the Equal Opportunity in Education Act was enacted in 1972, female sport participation increased dramatically from 3 % among high school athletics to 40 % in 2011 [1]. Female participation at youth high school and collegiate levels continues to grow. With the substantial rise in athletic participation, the many positive health benefits to women engaging in exercise have become apparent. However, a distinct set of health problems unique to the female athlete has also emerged.

The female athlete triad (Triad) was first defined in 1992 as a disorder involving three distinct but interrelated conditions including eating disorder, amenorrhea, and osteoporosis [2]. It was initially thought to most commonly affect women participating in esthetic and weight-dependent sports, including gymnastics, ice skating, and endurance running. Many athletes remained undiagnosed and without treatment because they did not meet the classic Triad criteria [3–5].

**Fig. 1:**
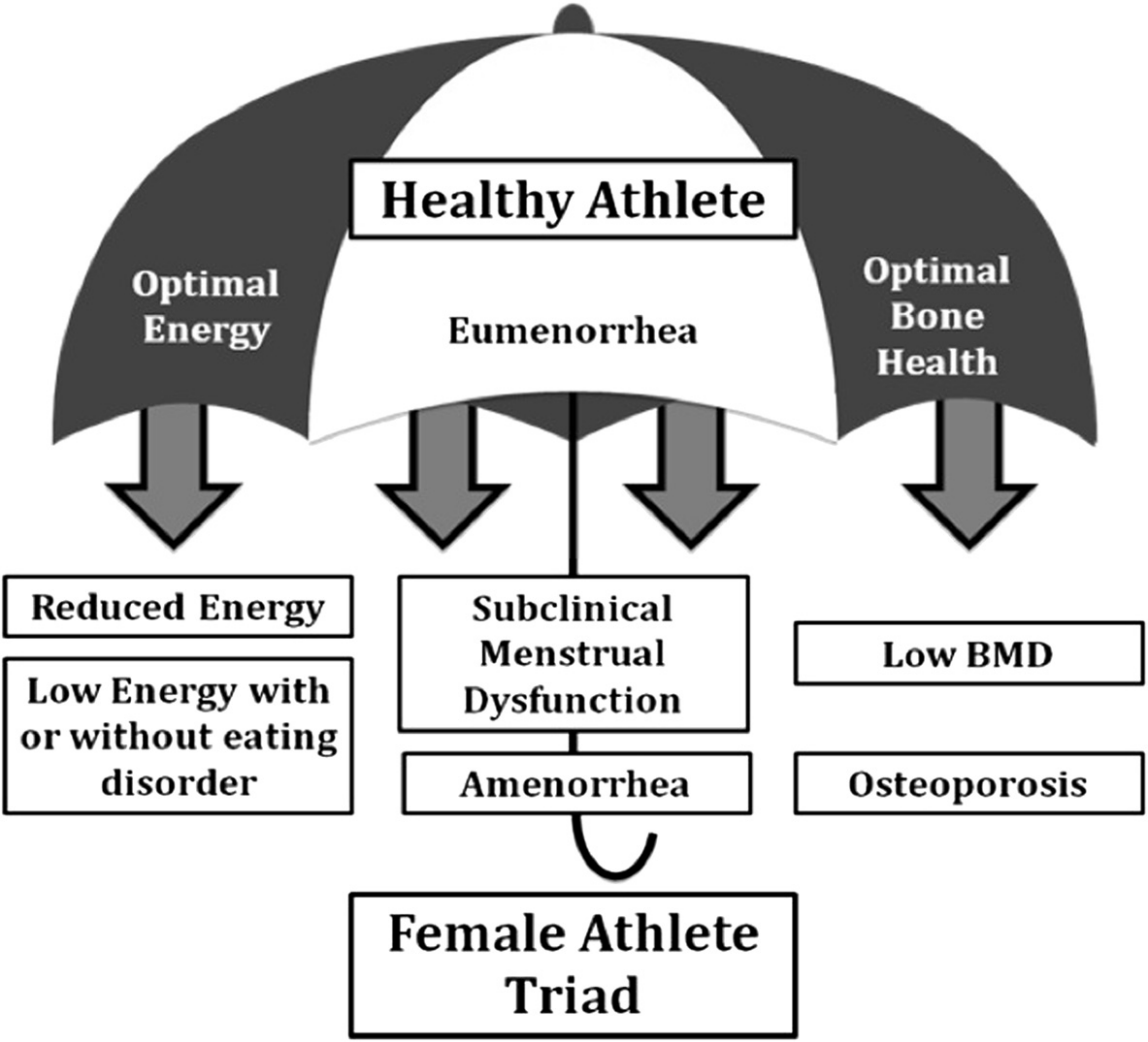
The female athlete triad is now defined as a spectrum disorder, so many female athletes may fit under the umbrella of Triad without having all three features of Triad concomitantly. *BMD* bone mineral density, *optimal energy* optimal energy availability, *reduced energy* reduced energy availability

The concept of the Triad was updated by the American College of Sports Medicine in 2007 in an effort to capture more athletes who may be at risk for the Triad health sequelae but did not fit the former definition. The female athlete triad is now considered a three-disorder spectra involving energy availability (EA), menstrual function, and bone health ranging from a healthy status to subclinical and clinical endpoints [6]. Physicians must be aware that many athletes can fit under the Triad “umbrella” without having all three poor clinical components of Triad simultaneously (Fig. 1) [7].

There is a wide variability in studies that investigate the prevalence of the Triad, such as patient population, sample size, and Triad utilized [8]. One study reported disordered eating, menstrual dysfunction, and low bone mineral density (BMD) accounted for 18.2, 21.8, and 23.5 % of high school athletes, respectively [4]. The Triad can be a debilitating disorder with both short-term and lifelong consequences for the female athlete if left undiagnosed. Sustained low EA associated with the Triad can impair health and wellness, causing medical complications that can impact the skeletal, endocrine, cardiovascular, reproductive, and central nervous systems [9]. In fact, the International Olympic Committee has gone a step further to suggest that the Triad is one important part of a constellation of health and performance consequences of low EA, having recently coined an umbrella term of “relative energy deficiency in sport (RED-S)” [10].

Despite expanding the definition of the Triad in 2007, and updates in Triad and RED-S screening from the Female Athlete Triad Coalition and the International Olympic Committee in 2014 and 2015, awareness seems to remain low among coaches, trainers, and clinicians directly involved in the care of female athletes, with a paucity of literature addressing the Triad knowledge of these groups [6, 10–12]. The most recent studies addressing the knowledge of these groups published in 2006 had small sample sizes and demonstrated poor Triad awareness [13, 14]. Additionally, while awareness on the part of coaches and trainers is imperative as they are often on the frontline with athletes, it is typically the physician who has the authority to order various medical tests, make the diagnosis, and coordinate care of the affected athlete with an appropriate treatment team. Thus, it is paramount that physician awareness be assessed and maximized. Physicians often make the call when it comes to withdrawal and return to play decisions. However, there is little research investigating Triad awareness across medical specialties at different levels of training [13]. Additionally, there is little research investigating awareness across medical specialties at different levels of training. This is concerning as identification, early intervention, and prevention are necessary to avoid the more serious negative clinical sequelae of the Triad. Therefore, our primary aims of this study were to (1) determine the percentage of physicians (at various levels of medical training across specialties) who are aware of the Triad and (2) determine the percentage of who can properly diagnose or refer patients to appropriate specialists.

## Methods

**Fig. 2:**
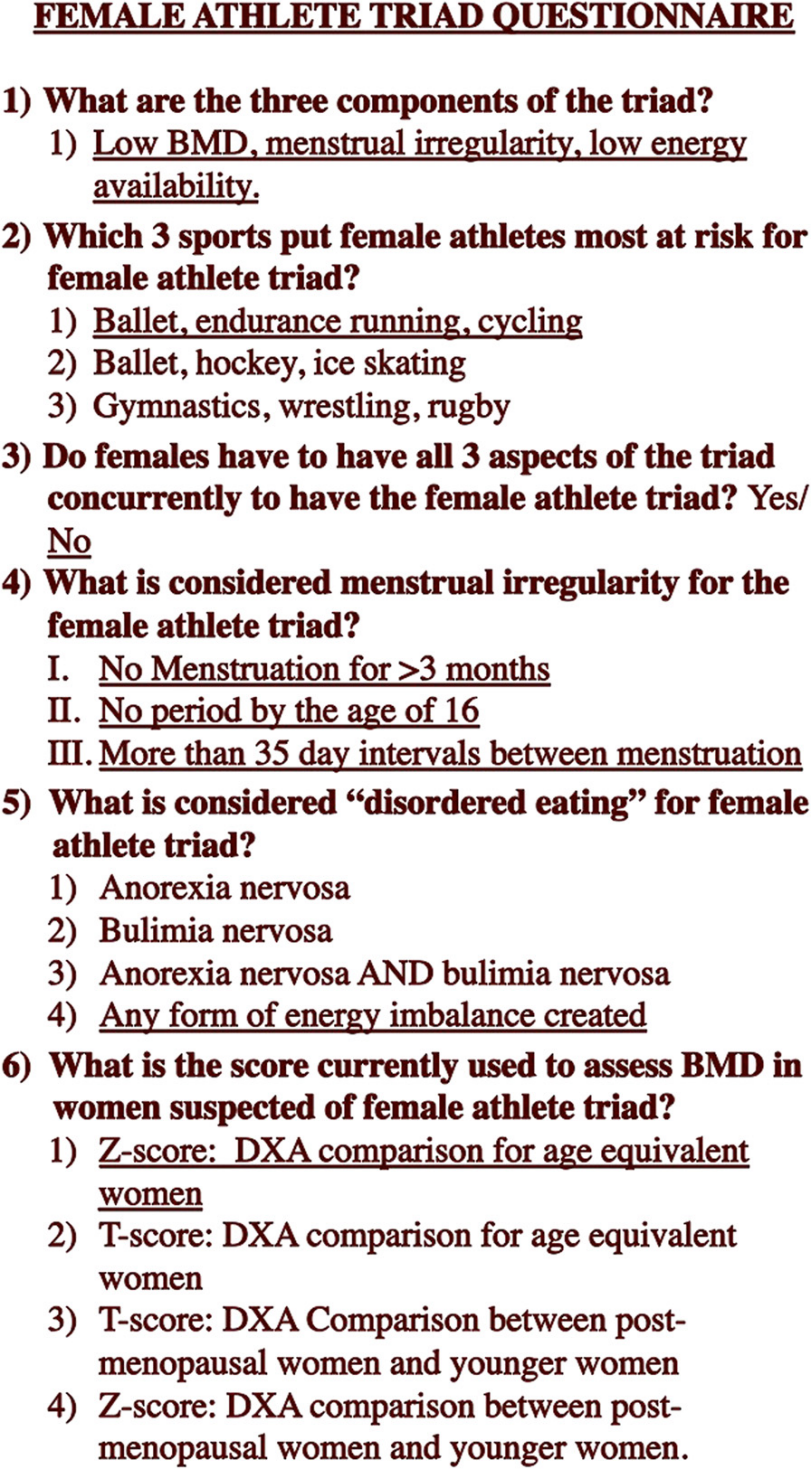
Female athlete triad awareness questionnaire administered. The correct answer to each question is underlined. *BMD* bone mineral density, *DXA* dual energy X-ray absorptiometry

We recruited medical staff, fellows, and residents at three large tertiary care centers and academic institutions across specialties via electronic survey to answer an 8-item test on Triad awareness and knowledge (Fig. 2). Question 1 was a fill-in-the-blank response, and one point was given for proper identification of each Triad component. Question 4 had check boxes for response when multiple choices could be selected (respondents were told this as a subheading within the survey). The survey was written by investigators and piloted among residents, physician assistants, and fellows within the department of orthopedics. The questionnaire first asked if the physician had heard of the female athlete triad. If the physician answered “no,” the questionnaire ended. If the physician answered “yes,” then the questions proceeded to assess the ability to properly identify the three basic components of the Triad, basic knowledge about each component, and comfort in the ability to treat or refer a patient presenting with one or more component of the Triad.

REDCap Data Collection software was used for survey administration via e-mail. The survey was sent to approximately 3961 physicians at three different academic teaching hospitals. The survey was sent out using the central hospital e-mail list comprising e-mail addresses for physicians, fellows, and residents for two institutions. The third institution agreed to send the survey via the hospital newsletter as an optional survey with a link. One subsequent reminder e-mail was sent to those who did not initially respond to the survey to increase response rate. Responses from a total of 931 physician participants were recorded and categorized according to the following specialties: anesthesia, emergency medicine, medicine, neurology, obstetrics/gynecology, orthopedics, physiatry, psychiatry, radiology, rheumatology, and surgery. Medicine was comprised of allergy/immunology, cardiology, critical care, dermatology, endocrinology, gastroenterology, family medicine, general internal medicine, infectious disease, nephrology, pediatrics, sleep medicine, and urology. Surgery was comprised of burns surgery; cardiac surgery; ear, nose, and throat surgery; gastrointestinal surgery; general surgery; neurosurgery; otolaryngology; pediatric surgery; plastic surgery; transplant surgery; and trauma surgery. Responses were given a score based on an eight-point scale total with the following breakdown: one point awarded for each properly answered multiple choice question and three points awarded for properly identifying the three components of the Triad.

Analysis was then performed using SAS Version 9.3 software. For the univariate analyses, independent *t* tests were used to test statistical significance when comparing two groups, while analysis of variance (ANOVA) methods were used for multiple group comparisons. To compare whether there was a difference between genders as well as among hospitals regarding ever having heard of the Triad, a Pearson chi-square analysis was performed. To investigate differences further, a logistic regression model was created and provided odds ratios of differences, which were also compared. A Mantel-Haenszel chi-square test was performed for comparisons involving test scores. This study was approved by the Partners Human Research Committee (Partners IRB) and Committee on Clinical Investigation (Boston Children's Hospital IRB) and has been performed in accordance with the ethical standards of the Declaration of Helsinki.

## Results

We had a response rate of 23.5 % across three large academic hospitals. Of the total respondents (*n* = 931), 40 % (*n* = 373) were male and 60 % (*n* = 558) were female. Twenty-three percent were residents (*n* = 214), 12 % were fellows (*n* = 112), and 65 % were attending physicians (*n* = 605) (Table 1). The average number of years in practice for attending physicians was 15 ± 11 year, and years in residency for residents was 2.5 ± 1.2 years. Overall, only 37 % (*n* = 343) had heard of the Triad. Of these respondents, an average of 2.1 ± 1.1 of the three components were properly identified with an overall average score on the Triad awareness test of 71 ± 18 % out of a possible 100 %. Of the physicians surveyed, 51 % reported feeling comfortable treating or referring a patient with the Triad, while 49 % did not.

**Table 1:** Survey response rate based upon level of training and specialty

Specialty	Resident	Fellow	Attending
Medicine	107	67	295
Surgery	15	4	48
Ortho	21	10	30
Rheum/PM&R	9	2	16
Radiology/pathology	5	6	41
Anesthesia	32	5	52
ER	0	5	31
Psychiatry	19	5	21
Neurology	6	6	23
Ob/gyn	0	2	27
Total	214	112	584

The breakdown of awareness based on level of training demonstrated that 32 % of the attending physicians (*n* = 110), 46 % of the fellows (*n* = 158), and 44 % of the residents (*n* = 151) had heard of the Triad. The likelihood of a fellow having heard of the Triad was 1.8 times that of an attending (*p* < 0.006), while the likelihood of a resident having heard of it was 1.6 times that of an attending (*p* < 0.003). Interestingly, the overall score on the questions did not differ among the three training levels (*p* = 0.89); however, the residents and fellows were significantly better at properly identifying the three components of Triad compared to attending physicians (*p* < 0.007). The attending physicians and fellows reported feeling more comfortable than the residents in treating/referring patients with the Triad (55 and 56 % vs. 38 %).

**Fig. 3:**
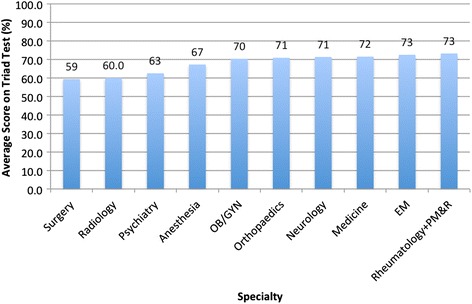
The percentage of respondents who have heard of Triad based on specialty. *OB/GYN* obstetrics and gynecology, *EM* emergency medicine, *PM&R* physical medicine and rehabilitation

**Fig. 4:**
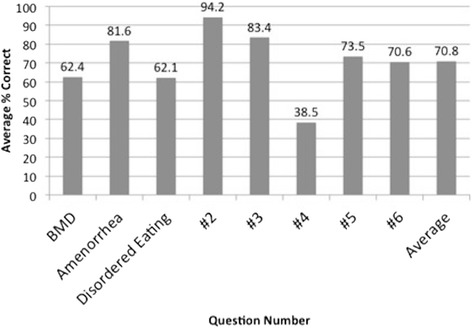
The overall percentage of each question answered properly with bone mineral density, amenorrhea, and disordered eating (including properly naming the components of the Triad from question number 1). *BMD* bone mineral density

When assessing awareness among specialties, the awareness rates were highest among orthopedics (80 %), followed by obstetrics and gynecology (55 %) and physical medicine and rehabilitation/rheumatology (52 %). The three specialties with the lowest awareness were anesthesia (9 %), radiology (10 %), and psychiatry (11 %) (Fig. 3). There were no significant differences between physician genders regarding awareness of the Triad, score on identifying the three components, or overall score (Fig. 4).

## Discussion

Awareness of Triad was first assessed in a study published by Troy and colleagues in 2006 [13]. The researchers found low awareness among the 240 athletic trainers, coaches, medical students, physical therapists, and physicians surveyed. Forty-eight percent of physicians and 32 % of medical students were able to identify the Triad. A mere 9 % of physicians surveyed felt comfortable treating a patient with the disorder. When looking exclusively at pediatricians, 36 % of pediatricians were able to identify the Triad components and only 4 % felt comfortable treating the disorder. This study was limited by sample size [13]. A more recent study from 2014 assessed the ability of 370 US high school nurses to diagnose the three components of the Triad and found that only 19 % were able to do so, but 95 % expressed interest in learning more about the disorder [15].

In a 2006 study assessing coaches’ awareness of the Triad, approximately 43 % of coaches could properly identify the three components, yet only 8 % of coaches reported always assessing menstrual function prior to sport participation [14]. This study had limitations, as well. Most respondents were female, which is not representative of typical coach demographics. Additionally, the response rate was only 30 %, and the sample size was low.

Our findings suggest that slightly more than one third of the residents, fellows, and attending physicians surveyed from the three academic centers have heard of the female athlete triad. Those aware of the Triad scored 71 ± 18 % of a possible 100 % on our questionnaire, which consisted of basic knowledge necessary to properly treat/refer this at risk population. Current residents and fellows have increased odds of having heard of it and properly identifying the three components compared to attending physicians; however, there was not a statistically significant difference in overall awareness score among training levels.

Increasing awareness among health-care providers across specialties is paramount, as one of the primary challenges with the Triad is difficulty in identification. Awareness of the Triad and its components is necessary in order to encourage behavioral changes in these patients. The 2014 Female Athlete Triad Coalition Consensus Statement [11] endorses screening for the Triad as a portion of the preparticipation physical evaluation (PPE) [16–19]. The consensus statement also presents the ‘Female Athlete Triad: Cumulative Risk Assessment’ to provide an objective method of determining an athlete’s risk for the Triad using risk stratification and evidence-based risk factors. Additionally, the International Olympic Committee has recently published a RED-S Clinical Assessment Tool (RED-S CAT) to help identify athletes at risk of low EA. [12] Although there is limited evidence regarding the efficacy of such screening tools, the current standard screening PPE form includes nine questions related to the Triad [20]. Screening is most typically completed for female athletes at the collegiate level but should be done at the high school level as well [11]. This younger age group should be targeted for three main reasons: (1) the declining age of onset of eating disorders; (2) the opportunity to maximize bone accrual during adolescence (greater than 90 % of peak bone mass is attained by 18 years of age with the greatest rate bone mass accrual occurring between ages 11 and 14); and (3) the earlier Triad components are detected, the better they will respond to treatment [21, 22].. Therefore, adolescent female athletes may represent the most important population to target.

Providers should be aware that most patients will only present with one or two components of the Triad, but may be at risk for all three. Schtscherbyna et al. [23] conducted a study in 2009 assessing 78 elite swimmers aged 11–19 years and found that 44.9 % met the criteria for disordered eating, 19.2 % for menstrual irregularity, and 15.4 % for low bone mass. Nichols et al. [4] found that out of 170 high school female athletes, 18.3 % met the criteria for disordered eating, 23.5 % for menstrual irregularity, and 21.8 % for low bone mass. However, of these athletes, only 5.9 % had two components and 1.2 % had all three. Focusing on the three spectra concept of the Triad in future education initiatives will be crucial to optimal physician diagnosis and treatment planning, as many studies have shown that athletes often present with variable severity of the individual components [4, 23, 24].

It is discouraging that our study demonstrates that less than half (47 %) of medicine physicians (including pediatricians, internists, and family medicine physicians) are aware of the Triad. Such physicians should feel comfortable screening and initiating a work-up for each Triad component and potentially developing a multi-disciplinary treatment approach. These medical providers are the ones most often involved in annual health screening, and they should be familiar to Triad risks and consequences when evaluating their athletic patients. Primary care sports medicine physicians are frequently involved in high school and collegiate athlete team coverage. Because there were so few primary care sports medicine physician responders, Triad awareness and knowledge of this subgroup of clinicians was not specifically identified in our survey.

Psychiatrists and psychologists are frequently involved in treatment of female athletes with disordered eating and the mood and anxiety dysfunction that frequently affects females along the Triad spectra. However, this specialty had the poorest Triad awareness in our study. Education should be particularly emphasized in both psychiatry and psychology training programs to help promote early recognition and management of female athletes with the Triad.

It is encouraging that Triad awareness was relatively high among orthopedic surgeons (80 %), as this group of physicians is also frequently involved directly in athletic team coverage and the care of female athletes in clinics and training rooms. Orthopedists who evaluate and treat females for bone stress injuries should be comfortable questioning such patients about the other components of the Triad (e.g., dietary patterns and menstrual history). Ideally, Triad patients are identified prior to the development of stress fracture, which can be a result of low EA, menstrual dysfunction, and decreased bone density [25, 26].

There are limitations to this study. Due to sample size and inconsistent reporting of specific specialties, we were required to combine several specialties for statistical analysis purposes. For example, the “medicine” group includes specialties such as family medicine, pediatrics, general internal medicine, internal medicine specialties, and any of the above who may have also completed a primary care sports fellowship. This survey was administered to three academic teaching hospitals in a large city, which may not be representative of the level of awareness among community physicians at non-academic institutions. Additionally, we had a low response rate of just 23.5 %, questioning the extrapolation of our results to greater populations of clinicians. The survey was e-mailed from an academic attending at each teaching hospital to the attending physicians and house staff of their respective hospitals, with a clear heading of its intent “Survey of Female Athlete Triad Awareness”, with a follow-up reminder e-mail as well. Thus, it is quite likely that many of those with an awareness, interest, or concern for the topic responded. We suspect this would, if anything, result in our over-reporting awareness of the Triad among medical specialties.

## Conclusions

It is clear that a multi-disciplinary teams of clinicians, coaches, and other health and athletic professionals need to work together to heighten awareness of the Triad. This appears to be the best approach to capture Triad athletes early to engage in prevention and treatment strategies. This multi-disciplinary approach is also favored for athletes diagnosed with Triad to foster appropriate management of the interrelated components.

Given the low awareness of Triad and its elements among health-care professionals, efforts should be made to educate the medical community early by integrating Triad teaching into medical school curricula and residency education. Continuing efforts to conduct and publish research on this important topic in sports medicine will raise further awareness.
